# Focal endoscopic intermuscular dissection guided by the pocket-detection method for radical excision of early T2 rectal cancer

**DOI:** 10.1055/a-2615-5775

**Published:** 2025-07-02

**Authors:** Andrea Sorge, Maria Eva Argenziano, Michele Montori, Pieter Jan Poortmans, Anne Hoorens, Gian Eugenio Tontini, David James Tate

**Affiliations:** 1 9304 Department of Pathophysiology and Transplantation, University of Milan, Milan, Italy; 2 60200 Department of Gastroenterology and Hepatology, University Hospital Ghent, Ghent, Belgium; 3 9294 Clinic of Gastroenterology, Hepatology and Emergency Digestive Endoscopy, Università Politecnica delle Marche, Ancona, Italy; 4 60201 Department of Gastroenterology and Hepatology, University Hospital Brussels (UZ Brussels), Brussels, Belgium; 5 60200 Department of Anatomopathology, University Hospital Ghent (UZ Gent), Gent, Belgium; 6 Gastroenterology and Endoscopy Unit, Fondazione IRCCS Ca’ Granda Ospedale Maggiore Policlinico, Milan, Italy; 7 26656 Faculty of Medicine and Health Sciences, University of Ghent, Ghent, Belgium

An 80-year-old man presenting hematochezia was referred to our institution due to a 20 mm slightly elevated rectal lesion with a central depression (Paris 0–IIa+c) on the right-posterior rectal wall below the inferior Houstonʼs valve ( Video 1 ). The macroscopic appearance and virtual chromoendoscopy (JNET III surface and vascular pattern) suggested a deeply invasive cancer ( Fig. 1 ). Staging pelvic magnetic resonance imaging revealed rectal cancer with invasion but partial preservation of the muscularis propria (T1b/early T2) without malignant lymph nodes or extramural vascular invasion. A total body computer tomography (CT) scan did not reveal distant metastases. After a multidisciplinary team discussion, the patient refused total mesorectal excision, and an endoscopic local excision was then offered.

Focal endoscopic intermuscular dissection achieving a radical resection of an early T2 rectal cancer.Video 1

**Fig. 1:**
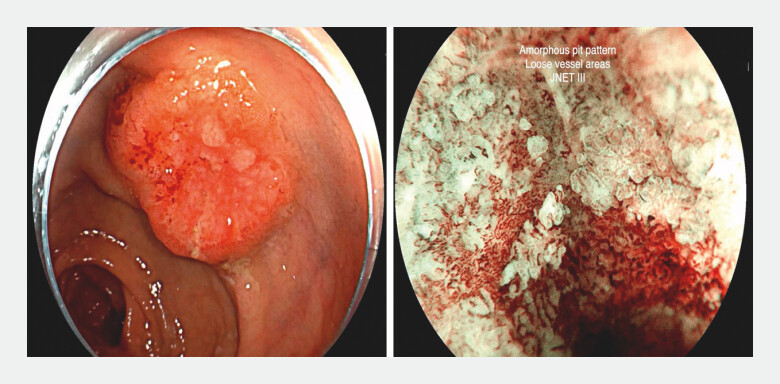
White light appearance (left) of a Paris 0–IIa+c lesion, 20 mm in diameter, located on the right-posterior wall of the distal rectum. Virtual chromoendoscopy (right) was suggestive of deeply invasive cancer (amorphous surface and loose vessel areas, Japan NBI Expert Team [JNET] classification III).

Creating a submucosal pocket towards the deeply invasive component (pocket-detection method 1 2 ), the muscle-retracting sign indicating the deeply invasive area within the lesion was identified ( Fig. 2 ) and circumferentially isolated 3 . Following multiband-and-wire pulley traction 4 application, incision of the circular layer of the muscularis propria was performed around the suspected invasive component at a safety distance of 3 mm to achieve R0 while minimising the intermuscular dissection area ( Fig. 3 ). The focal endoscopic intermuscular dissection (EID) was completed without complications, and the patient was discharged 24 hours after the resection. Histopathology ( Fig. 4 ) revealed a radical resection of a well-differentiated adenocarcinoma invading the muscularis propria without lymphovascular invasion or tumour budding (pT2). Given the radical resection and the patientʼs age and preference, the multidisciplinary team agreed on a follow-up. At the 3-month follow-up, there was no endoscopic recurrence or functional impairment, and the total body CT scan revealed no distant metastases.

**Fig. 2:**
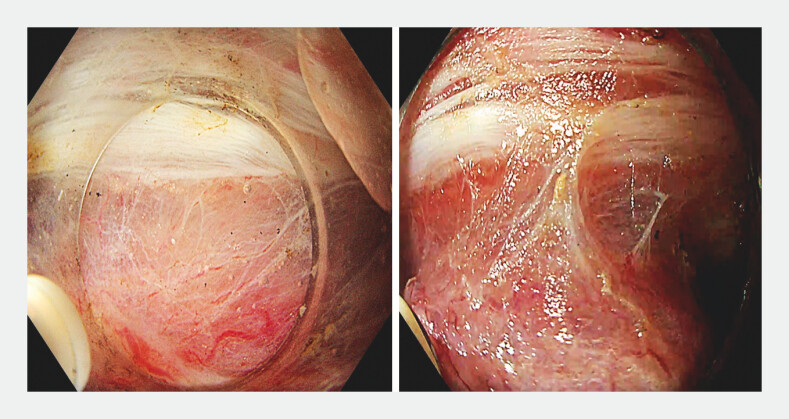
Muscle-retracting sign. Submucosal invasion and fibrosis causing tethering of the muscularis propria to the overlying lesion, narrowing of the submucosal space, and non-staining submucosa. Appearance under saline immersion (left) and CO _2_ insufflation (right).

**Fig. 3:**
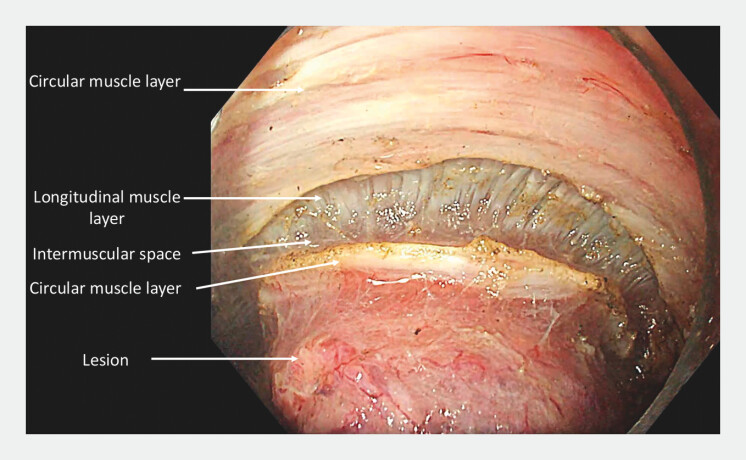
Appearance of the resection defect following the circumferential incision of the circular layer of the muscularis propria. The submucosal and intermuscular dissection planes are exposed by the multiband-and-wire pulley traction.

**Fig. 4:**
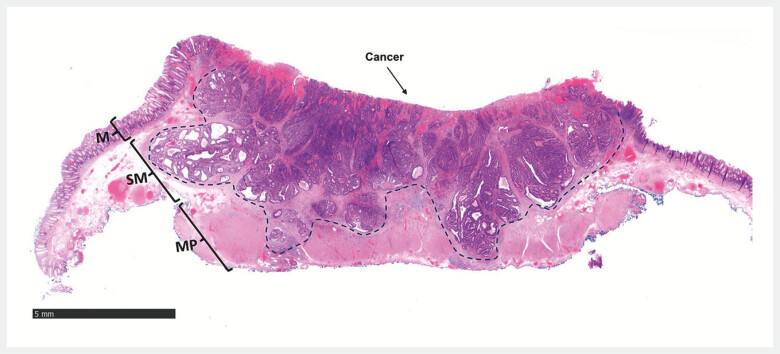
Tissue section (hematoxylin and eosin staining) of the rectal endoscopic resection specimen showing a low-grade adenocarcinoma invading deep into the muscularis propria with margins free of neoplasia. M = mucosa, SM = submucosa, MP = muscularis propria. The dashed line marks the invasive front of the tumour.

The novel focal EID guided by the pocket-detection method enabled safe and R0 resection of a T2 rectal cancer in an elderly patient refusing surgery. Focal EID may decrease the area of circular muscular resection, potentially reducing procedural time and complication rates.

Endoscopy_UCTN_Code_TTT_1AQ_2AD_3AZ

## Notes

Endoscopy E-Videos https://eref.thieme.de/e-videos
*E-Videos* is an open access online section of the journal *Endoscopy* , reporting on interesting cases and new techniques in gastroenterological endoscopy. All papers include a high-quality video and are published with a Creative Commons CC-BY license. Endoscopy E-Videos qualify for HINARI discounts and waivers and eligibility is automatically checked during the submission process. We grant 100% waivers to articles whose corresponding authors are based in Group A countries and 50% waivers to those who are based in Group B countries as classified by Research4Life (see: https://www.research4life.org/access/eligibility/ ). This section has its own submission website at https://mc.manuscriptcentral.com/e-videos .
